# Lower Depression Scores among Walnut Consumers in NHANES

**DOI:** 10.3390/nu11020275

**Published:** 2019-01-26

**Authors:** Lenore Arab, Rong Guo, David Elashoff

**Affiliations:** 1 Division of General Internal Medicine and Health Services Research and Department of Biological Chemistry, David Geffen School of Medicine, Los Angeles, CA 90095, USA; 2 Department of Medicine Statistics Core, David Geffen School of Medicine, University of California, Los Angeles, CA 90095, USA; rongguo@mednet.ucla.edu (R.G.); delashoff@mednet.ucla.edu (D.E.)

**Keywords:** depression, nut consumption, NHANES, epidemiology, walnut

## Abstract

Background: Multiple studies have shown a Mediterranean diet, characterized by their olive oil and nut consumption, to be correlated with lower depression risk. Objective: To examine whether part of this reduced risk in the United States is attributable to walnut consumption, we analyzed data on walnut consumption and depression scores from the National Health and Nutrition Examination Survey (NHANES). Methods: NHANES survey data for 2005 through 2014 was pooled for adults with 24 h recall dietary data. Depression scores were based on PHQ-9 self-report responses. A total of 26,656 participants were characterized as reporting the consumption of walnuts with high certainty, walnuts with other nuts, other nuts, or no nuts. Results: After an adjustment for covariates, walnut consumers showed lower depression scores compared to non-nut consumers. The least square mean for total depression score was 26% lower for walnut with high certainty consumers than for non-nut consumers (*p* < 0.0001), and the association was stronger among women (32%, *p* < 0.0001) than men (21%, *p* = 0.05). The significant contributors to this difference were due to walnut consumers reporting greater interest in doing things (*p* = 0.003), less hopelessness (*p* = 0.02), and feeling more energetic (*p* = 0.05) than non-nut consumers. Non-nut consumers were more likely to have trouble concentrating (*p* = 0.02), to feel they were moving or speaking abnormally (*p* = 0.03), and to have thought they were better off dead (*p* = 0.002). Conclusions: Depression scores were significantly lower among nut consumers and particularly walnut consumers as compared to non-nut consumers. After controlling for potential covariates, walnut users had scores significantly lower than other nut consumers. The difference was strongest among women, who are more likely than men to report higher depression scores.

## 1. Introduction

Depression, especially among women, is a significant public health issue across the globe. A crude surrogate can be the estimates of persons 12 years of age or older who took antidepressants in the past month (12.7%). Among women in the United States the usage is almost twice as high as among men, approaching one in six women [1]. Across all age groups, antidepressant usage has increased 65% over the past decade in the US. Although antidepressant usage may be indicative of other underlying problems not just clinical depression, the depressive symptomatology of the population is of concern. The various risks related to depression range from suicide to obesity. Depression can both cause and be caused by poor diets. The percentage of people reporting depression in the US who are obese is 43% [1]. Understanding and addressing the possible role of poor diets in depressive symptoms can be of great public health importance.

Observational studies consistently find dietary patterns associated with depressive symptoms. This is shown in longitudinal studies in Japan, Italy, and the Netherlands [2]. Understanding which part of the diet may reduce depression risk can provide a low-cost public health intervention to reduce the incidence of this condition and its sequelae. In the population at large, interest is drawn to dietary patterns that promote health and well-being. Healthy eating patterns have been inversely associated with depression scores in the US [3]. Some of the strongest evidence of a dietary connection in humans comes from studies of people on a Mediterranean diet. As early as 2013, a pooled analysis across 9 studies showed a 32% lower likelihood of depression (RR 0.68, 95%CI = 0.54–0.86) among adherers to a traditional Mediterranean diet [4]. Since then, two additional intervention studies reported lower depression scores among subjects assigned to a Mediterranean diet [5,6]. In an open trial, a 60% reduction in the incidence of extreme depression among Australians reporting depression when on Mediterranean diets [6]. Although the food sources responsible for these effects remain unknown, many Mediterranean diets, including both of those cited, are characterized by the relatively high consumption of nuts. In fact, both of these interventions, along with two recently published [7,8] cohort studies, have found higher nut consumption associated with lower depression scores. 

Tree nuts, often characterized by healthy fatty acid profiles, differ substantially in their nutritional profiles, with walnuts being high in alpha-linolenic acid, a fatty acid associated with brain health in animal studies [9]. In addition, the polyphenol content of walnuts differs from other tree nuts in ways that might affect the gut–brain axis related to serotonin production [10]. Walnuts have also been associated with positive effects on cognition [11]. To further explore the association between nut consumption, and in particular walnut consumption, and depression, we employed data from the National Health and Nutrition Examination Survey (NHANES) from 1999–2014 [12]. This data was used to discern the relationship between walnut consumption and depression, as compared with other nuts and non-nut consumption in the population at large.

## 2. Methods

### 2.1. Participants

NHANES Sample. The National Health and Nutrition Examination Survey (NHANES) is a cross-sectional, probability survey administered to the US civilian population aged from 0 to 85 years. This study used continuous NHANES data collected from 2005 to 2014, covering 5 cycles of data collection (*N* = 50,965). Participants were excluded from analysis for being under 18 (*N* = 21,737). Covariates were derived from the data files series _D through _H for the demographic data files “Demo-”, depression data from the mental health questions in “DPQ_”, alcohol and smoking data from “ALQ_” and “SMQ-”, and the dietary data from DR1TOT_D through HData and DR2TOT_D through _HData. In an attempt to have a control group free of nut consumers, individuals who consumed peanuts but not other nuts on the day of recall were also excluded from the analysis. Peanuts were not included as they are not tree nuts. Also missing are participants lacking data on either day of the 24 h food intake (*N* = 2572). After exclusions, our analytic sample N was 26,656, representing a population size of approximately 225 million individuals. All analyses were weighted using a 10 year sampling weight created from the day 1 24 h dietary recall weights in accordance with the NHANES Analytic and Reporting Guidelines.

A subset of adults conducted repeat 24 h recalls by telephone 3 to 10 days after the baseline dietary assessment. A high level of concordance was noted (~82%) in the walnut consumption classification between both days.

### 2.2. Measures

Dietary Intake Interview: Dietary intake interviews were conducted in-person in the NHANES mobile examination clinics. The specific types of food as well as the amount of foods consumed the previous day were recalled by survey participants. To help in assessing the amount of food consumed, recall cue items such as food charts, measuring cups, and rulers were provided. Survey participants were requested to recall all foods and beverages consumed during the past 24 h from midnight to midnight. A second dietary recall was repeated in a subset of individuals. In order to improve the identification of walnut consumers, data from both days were used such that participants reporting walnut consumption in either interview were classified as consumers and gram amounts were averaged if walnut consumption was reported on both days. 

The NHANES dietary data applies USDA food codes. These were used to identify foods that described or included walnuts by searching for the word “walnut” in the food code label descriptors and to identify foods that described or included other tree nuts by searching for the words “nut”, “almond”, “macadamia”, “alpine”, “cashew”, “pecan”, “pistachio”, and “granola”. 

Walnut Consumption Groups: The food codes used to identify walnut consumption are as in our previous publication on walnut consumption and cognition in NHANES [10]. Foods consisting entirely or mostly of clearly identifiable walnuts were classified as “walnuts with high certainty” (WwHC), and food codes describing nut mixes and foods that are likely to include walnuts but also other nuts were classified as “walnuts with other nuts” (WwON). In doing this, 2 levels of confidence about walnut consumption were created, the latter being diluted by other tree-nuts. The estimated amounts of walnuts consumed were totaled per individual across all foods consumed on that day. The amount assigned to walnut intake for mixed foods was calculated as a percentage of the reported amount based on the decomposition of recipes for the mixed food items, for example, for the amount of walnut in walnut muffins or the amount of walnuts in the mixed nuts reported. Food codes describing tree-nuts that are not walnuts (i.e., cashew nuts, pistachios, etc.) or nut-containing foods that do not commonly contain walnuts (e.g., granola) were classified as “other nuts” (ON) to allow for comparisons of walnut associations with other nut associations. All other foods that did not include walnuts or other nuts were classified as “no nuts” (NN). Participants who consumed at least one WwHC food were categorized to the WwHC group. Participants who did not indicate eating any nut-containing foods were classified as members of the no nut, NN group. 

Depression Outcomes: Participants were assessed for depression based upon their self-ratings on a PHQ-9 questionnaire for symptoms within the prior 2 weeks. This questionnaire is considered a valid and reliable instrument that is widely used to assess prevalence and severity of depressive symptoms [13]. Nine questions were scored on a scale of 0–3 for each frequency question of how often the symptom arose, with zero equaling not at all, 1 referring to several days, 2 referring to more than half the days, and 3 referring to nearly all days. The sum across all 9 questions was used as a quantitative measure of depression along with the cutoff of ≥10 as applied elsewhere [13]. Scores ≥5 were used to categorized individuals with mild depressive symptomatology.

Covariates: Covariates used in the adjusted analyses were age, sex, race, body mass index, alcohol consumption, smoking, annual household income, and marital status. The upper bound for the age data collected was 85+ years for NHANES cycle 2005–2006 and 80+ years for NHANES cycle 2007–2014. For the purposes of this study, age was winsorized to 80 years to be consistent across all cycles used. 

Alcohol consumption was dichotomized using a cutoff of 2 drinks per day based on their report of the average number of drinks in the last 12 months. Annual household income was categorized into under $20k, $20k–$75k/over $20k, $75k+, and missing. Smoking status was dichotomized into smokers and non-smokers. Marital status was categorized into married, widowed, divorced, separated, never married, living with partner, refused, and do not know. Race was categorized into white, black, Hispanic, and other, where participants reporting as “Mexican American” were combined with other “Hispanic” reporters.

### 2.3. Statistical Analysis

Standard description statistics were reported for each walnut consumption group, mean +/− standard error for numerical variables and percent (*n*) for categorical variables. The associations of walnut consumption with depression outcomes were assessed using linear regression for the depression sum score and logistic regression for depression defined by each item in the depression questionnaire (self-report depression item ≥1) and depression sum score (depression sum score ≥2, ≥5, or ≥10). Models were further adjusted for the aforementioned covariates. For depression sum score, the least square means with 95% confidence interval are calculated for each walnut consumption group. In the logistic models, the effects of the walnut consumption groups were presented based on the comparisons to the no nut group and the odds ratios of depression with 95% confidence interval were calculated. All analyses were weighted using the 10 year NHANES sample day 1 dietary weights and utilized the NHANES strata and population sampling units to account for the complex survey design. All the data analyses were conducted in SAS version 9.4 (SAS Institute Inc., Cary, NC, USA). 

## 3. Results

### 3.1. Description of Sample 

Presented in Table 1 are characteristics of the 26,656 adult participants who had dietary recall data. These values reflect the application of the appropriate sample weights for the US population over this ten year time period, 2005–2014. Of the individuals with 24 h recalls (23%) 6222 reported nut consumption, and of these, 3348 (54%) consumed walnuts. The average walnut consumption was 0.9 g/day across the entire population and 4 g/day for walnut consumption among the WWON consumers. The subset of individuals with specific walnut consumption, WWHC, consumed an estimated 24 g of walnut per day. Both other nut and walnut consumers tended to drink less alcohol, smoke fewer cigarettes, have a lower BMI, be older, were wealthier, and were more likely to be married than non-nut consumers. The consumption of walnuts is greater among women and Caucasians as compared with other races and ethnicities. 

### 3.2. Associations of Walnut Consumption with Depression Scores 

The depression status of the population by nut consumption is presented in Table 2. The unadjusted scores for each of the 9 contributing questions and their sum show trends of lower scores (less depression) among those consuming walnuts with other nuts and walnuts with high certainty. Depression sum scores were 45% lower among the WWHC consumers compared to NN consumers, with an average depression score of 3.3 for NN consumers versus 1.8 for those consuming WWHC and 2.6 for ON consumers. In addition to lower depression scores on the average, the prevalence of clinical depression defined as a total score greater than or equal to 10 was only one-third among WWHC (3%) and two-thirds among ON consumers (6%) as compared with NN consumers (9%). When the median score of 2 was used as a cut off, 12% more WWHC consumers were in the low scoring group than non-nut consumers (47% vs. 59%). There was also a 7% gap between the WWHC consumers as compared to WWON consumers (52% vs. 59%).

The results of the multivariable logistic and linear regression models of depression and walnut consumption can be found in Table 3. The least square means of total depression score and odds ratios of depression as a function of walnut consumption after applying sample weight, accounting for complex sample design and adjusting for covariates conventionally associated with depression (age, sex, race, BMI, smoking, alcoholic beverage consumption, and marital status) are reported. The least square means from multiple regression models show that each category of nut consumption was associated with lower depression scores than NN consumers. The least square means for the total depression score were 3.82 for WWON consumers as compared to 4.15 for non-nut consumers. For those consuming walnuts with high certainty, the effect was the strongest: 3.28 versus 4.15, which is only 79% of the NN least square mean score (*p* < 0.0001). This finding held for the gender specific analyses. Although both men and women consuming walnuts showed lower depression scores, the difference was greatest and significant among women, with a full point difference (3.54 WWHC v. 4.66 NN). 

Clinical depression based on depression sum scores of greater than 10 were less likely among consumers of WWON (odds ratio 0.67, 95% CI 0.48–0.93, *p* = 0.02) but not for the other groups, as compared with NN consumers, with this effect being driven by women. The most consistent effects were seen at the cutoff of 5 or more, consistent with mild depressive symptomatology. The likelihoods were significantly lower for ON (OR = 0.86), WWON (0.83) and were lowest among WWHC consumers (OR = 0.39 range 0.22–0.71).

An examination of the individual questions that contributed most significantly to this overall effect shows that six questions were independently associated with WWHC compared to NN consumption: little interest in doing things (*p* = 0.003); feeling down, depressed, or hopeless (*p* = 0.02); feeling tired or having little energy (*p* = 0.05); having trouble concentrating (*p* = 0.02); moving slowly (*p* = 0.03); and feeling one would be better off dead (*p* = 0.002). There appeared to be no significant association with poor appetite, trouble sleeping, oversleeping, or feeling bad about yourself. Gender stratified results show that among these adjusted analyses of individual questions, for those with significant overall effects, all effects were greatest among women, except for the question involving trouble concentrating on things.

Direct comparisons of walnut consumers with other nut consumers, as presented in Table 4, demonstrate that the walnut consumer had significantly lower depressive symptom scores after covariate adjustments than other nut consumers. The adjusted difference averaged 0.5 least square points across both genders. Mild symptomatology at the cut off of scores of 5 were also less frequent among walnut as compared with other nut consumers. This suggests that the walnut association is not an artifact of bias relating to nut consumption in general. A direct comparison of walnut consumers with other nut consumers shows them to have significantly lower depression scores.

## 4. Discussion

We found that in the US population, reported nut and walnut use, after controlling for covariates, remains significantly associated with lower prevalence and frequency of depressive symptoms. The mean depression scores of individuals, even in the normal range, are lower among the walnut consumers. We have also identified the components of depression reported to be less common among walnut consumers. These include greater interest in activities, higher energy levels, less hopelessness (among women), better concentration, and greater optimism. Nut consumption in general appears advantageous. We found this association, however, to be significantly higher among walnut consumers than consumers of other tree nuts. There are many significant differences noted between consumers and non-consumers of nuts and walnuts as noted in Table 1. We have attempted to control for these covariates, but other differences in diets may be influential in these findings. Sorting this out would require a randomized controlled feeding trial.

A growing understanding of the brain–gut connections is revealing pathways by which food choices may affect mood. A number of studies have focused on specific vitamins [4,14,15,16] and minerals [17]. A few have looked at individual fatty acid families [18,19,20]. However, in the population at large, interest is drawn to dietary patterns that promote health and well-being. Healthy eating patterns have been inversely associated with depression scores in the US [2]. A recent meta-analysis of all available observational studies (20 longitudinal and 21 cross-sectional) studies on healthy diets and risk of depressive outcomes from around the globe reported the most compelling evidence to be for the Mediterranean diet and incident depression from 4 longitudinal studies [21]). A systematic review restricted to prospective studies of diet quality and depression found consumption of healthy, prudent, and Mediterranean diets to be associated in dose-response fashion to depression symptoms as well [22]. An examination of the effects of nuts, as part of such diets, was inhibited by the lack of published reports. In this review, only two studies were identified that studied “nuts, seeds or soy”, and although the odds ratio was in a protective direction, at 0.92 with a range of 0.83 to 1.02, it was not significant. 

NHANES data has been used in a few publications of diet and depression previously. One of them used serum carotenoid levels as biomarkers of vegetable consumption and found significant trends among women but not men, using PHQ-9-determined symptomatology [14]. Just as depression and the use of anti-depressives are significantly greater among women than men, the magnitude of the effect of walnut consumption is greater among women than among men in our findings. The reasons for this remain unknown. This may be due to different doses or different risk profiles in general affecting background risk in women. It is well-known that women are more likely to report depressive symptomatology as compared with men [23]. We and others have often noted differences in effects of foods by gender. When studying cognitive function over time, for example, we found tea consumption slowed decline significantly more among women, and this was due to a more rapid decline in general among women than men [24]. Similarly, since depressive symptoms are greater among women, there may be more valence in ameliorating this among women. Alternatively, this may be a dose effect in which women consume more walnuts, but the power to examine this is not available in this dataset due to the relatively small number of walnut consumers.

Factors proposed by others as mechanisms for diet–depression associations relate to potential diet induced effects on oxidative stress, insulin resistance, inflammation, or altered vascularization [25]. Nuts have been directly hypothesized to affect metabolism in ways consistent with depression [26]. However, this wide speculative range of mechanisms underlies the lack of proven mechanisms.

The limitations of these findings are couched in the usual concerns that arise in cross-sectional observational studies. Such studies do not provide causal evidence and are subject to the risk of diet changing after a diagnosis occurs in response to the condition. Thus, depression might change appetite and eating behavior, resulting in apparent associations that are not causal. These findings could arise if people falling into depression choose to consume fewer nuts for some reason. Findings from longitudinal studies help address this. However, there are also numerous covariates that are related both with greater health in general and nut consumption, ranging from alcohol and tobacco use to greater wealth. The literature on relationships between wealth and depression is mixed, some of it showing greater wealth associated with more depressive symptoms [27]. The concerns regarding hidden confounds enhance the desire for randomized trials to substantiate the suggested preventive associations.

Another issue is the magnitude of measurement error that occurs when a study does not account for intra-individual variability in intake. Twenty-four h recalls can provide strong evidence of dietary consumption for frequently consumed foods but are much weaker unless repeated, for sporadically consumed foods. In cultures where there is great dietary diversity, the intra-individual variability can parallel the inter-individual variability and thus mask any effect. In any case, measurement error is to be expected, and its impact is to reduce risk estimates. Thus, our findings, if unbiased, are likely to underestimate the underlying signal. The strengths of this analysis, which adds to prior observational analyses in specific populations from other countries, is that it represents the US population at large and includes the inherent diversity in socio-economic class and race across the US.

It should also be noted that the depression symptoms assessed in this study were of short duration (the past few weeks) and the scoring system used included levels that would be considered mild in intensity. Therefore, these associations are not necessarily reflective of clinically relevant depression. 

In conclusion, a consistent association was observed between the consumption of nuts, and walnuts in particular, with fewer and less frequent depressive symptoms in a representative sample of the US population over the course of the past decade. This association was consistent across both gender but consistently stronger among women than men. Lower depression scores among consumers of walnuts appear to be traced back to better concentration, higher energy levels, more interest in doing things, and greater self-control of rates of speech and movement. These depression-related characteristics are lower among nut and walnut consumers. The association is seen across the spectrum of depression scores, both as differences at average levels of depression and differences in the percent of people defined as clinically (≥10) or mildly (≥5) depressed. Thus, a subtle and consistent relationship between nut-, and in particular, walnut-consumption and mood independent of other depression related variables is noted in cross sectional studies of American men and women. 

## Figures and Tables

**Table 1:** Description of Walnut Consumption and Covariates.

Description	Categories	Totals (*n* = 26,656)	Not a Nut Consumer 77% (*n* = 20,434)	Other Nut Consumer 12% (*n* = 3277)	Walnuts with Other Nuts Consumer 9% (*n* = 2542)	Walnuts with High Certainty Consumer 1% (*n* = 403)	*p*-Value
Avg number of alcoholic drinks/day-past 12 months		1.9 +/− 0.04 (25563)	2.1 +/− 0.04 (19489)	1.7 +/− 0.05 (3193)	1.6 +/− 0.07 (2484)	1.3 +/− 0.1 (397)	<0.0001
Age		46 +/− 0.3 (26656)	45 +/− 0.2 (20434)	47 +/− 0.5 (3277)	50 +/− 1 (2542)	55 +/− 1 (403)	<0.0001
BMI		28.6 +/− 0.1 (26329)	28.9 +/− 0.1 (20164)	27.9 +/− 0.2 (3250)	28.4 +/− 0.2 (2518)	27.0 +/− 0.4 (397)	<0.0001
Total Walnut (g)		0.9 +/− 0.05 (26656)	0 +/− 0 (20434)	0 +/− 0 (3277)	3.9 +/− 0.1 (2542)	23.7 +/− 1.6 (403)	
WwHC Total Walnut (g)		11.7 +/− 0.8 (403)				11.7 +/− 0.8 (403)	
WwON Total Walnut (g)		5.2+/− 0.2 (2945)			3.9 +/− 0.1 (2542)	12.0 +/− 0.8 (403)	
Annual Household Income	Under $20k	13% (4753)	15% (4035)	7% (356)	8% (315)	6% (47)	<0.0001
	$20k–$75k/Over $20k	39% (10993)	40% (8575)	34% (1203)	40% (1058)	36% (157)	
	$75k +	25% (4742)	22% (3147)	31% (822)	32% (634)	44% (139)	
	Missing	23% (6168)	23% (4677)	28% (896)	20% (535)	14% (60)	
Avg number of alcoholic drinks/day-past 12 months	<2	55% (15073)	53% (11264)	57% (1946)	60% (1575)	69% (288)	<0.0001
	≥2	45% (10490)	47% (8225)	43% (1247)	40% (909)	31% (109)	
Marital Status	Divorced	11% (2742)	11% (2088)	12% (340)	10% (266)	11% (48)	<0.0001
	Living with partner	7% (1921)	8% (1580)	6% (198)	5% (124)	5% (19)	
	Married	54% (12928)	51% (9484)	60% (1784)	61% (1415)	67% (245)	
	Never married	19% (4986)	21% (3996)	17% (553)	16% (404)	8% (33)	
	Separated	2% (841)	3% (708)	1% (72)	1% (53)	1% (8)	
	Widowed	6% (2082)	6% (1617)	4% (196)	7% (220)	7% (49)	<0.0001
Race	Black	12% (5844)	13% (4825)	7% (507)	8% (456)	5% (56)	
	Hispanic	13% (6654)	15% (5407)	11% (725)	9% (472)	4% (50)	
	White	68% (11928)	65% (8569)	76% (1705)	77% (1398)	86% (256)	
	Other	7% (2230)	7% (1633)	6% (340)	6% (216)	5% (41)	
Sex	Female	51% (13384)	49% (9885)	56% (1873)	54% (1366)	66% (260)	<0.0001
	Male	49% (13272)	51% (10549)	44% (1404)	46% (1176)	34% (143)	
Smoker	No	78% (19895)	75% (14663)	86% (2731)	87% (2132)	92% (369)	<0.0001
	Yes	22% (5403)	25% (4608)	14% (432)	13% (335)	8% (28)	

Mean ± SE (*N*) is reported for numerical variables and % (*N*) for categorical variables. *p*-values are from ANOVA for numerical variables and Rao–Scott chi-square tests for comparisons of frequencies for categorical variables. Subjects from 2005–2014 (DPQ data collected during this time period) who are older than 18 and have dietary recall data were included in the table.

**Table 2:** Description of Depression Outcomes within Walnut Consumption Categories.

	Depression Score Cutoffs	Total	No Nuts	Other Nuts	Walnuts with Other Nuts	Walnuts with High Certainty	*p*-Value
Little interest in doing things		0.33 +/− 0.01 (24814)	0.36 +/− 0.01 (18944)	0.28 +/− 0.01 (3093)	0.27 +/− 0.02 (2399)	0.13 +/− 0.02 (378)	<0.0001
Feeling down, depressed, or hopeless		0.32 +/− 0.01 (24829)	0.35 +/− 0.01 (18957)	0.27 +/− 0.01 (3096)	0.25 +/− 0.02 (2399)	0.15 +/− 0.02 (377)	<0.0001
Trouble sleeping or sleeping too much		0.62 +/− 0.01 (24833)	0.65 +/− 0.01 (18959)	0.56 +/− 0.02 (3096)	0.55 +/− 0.02 (2400)	0.49 +/− 0.05 (378)	<0.0001
Feeling tired or having little energy		0.73 +/− 0.01 (24831)	0.76 +/− 0.01 (18957)	0.66 +/− 0.02 (3096)	0.67 +/− 0.02 (2400)	0.47 +/− 0.04 (378)	<0.0001
Poor appetite or overeating		0.35 +/− 0.01 (24836)	0.38 +/− 0.01 (18964)	0.28 +/− 0.02 (3095)	0.28 +/− 0.01 (2400)	0.27 +/− 0.04 (377)	<0.0001
Feeling bad about yourself		0.25 +/− 0.01 (24816)	0.26 +/− 0.01 (18946)	0.22 +/− 0.01 (3096)	0.21 +/− 0.01 (2397)	0.12 +/− 0.02 (377)	<0.0001
Trouble concentrating on things		0.25 +/− 0.01 (24827)	0.27 +/− 0.01 (18957)	0.21 +/− 0.01 (3095)	0.22 +/− 0.01 (2399)	0.11 +/− 0.02 (376)	<0.0001
Moving or speaking slowly or too fast		0.15 +/− 0.01 (24817)	0.17 +/− 0.01 (18945)	0.1 +/− 0.01 (3095)	0.11 +/− 0.02 (2400)	0.06 +/− 0.02 (377)	<0.0001
Thought you would be better off dead		0.05 +/− 0 (24820)	0.05 +/− 0 (18948)	0.03 +/− 0.01 (3096)	0.02 +/− 0 (2399)	0 +/− 0 (377)	<0.0001
Sum of Depression Item Scores		3.05 +/− 0.06 (24721)	3.25 +/− 0.07 (18861)	2.6 +/− 0.09 (3090)	2.58 +/− 0.1 (2395)	1.81 +/− 0.17 (375)	<0.0001
Sum of Depression Item Scores	<10	92% (22560)	91% (17062)	94% (2888)	95% (2252)	97% (358)	<0.0001
	≥10	8% (2161)	9% (1799)	6% (202)	5% (143)	3% (17)	
Sum of Depression Item Scores	<5	77% (18762)	75% (14090)	81% (2445)	81% (1902)	90% (325)	<0.0001
	≥5	23% (5959)	25% (4771)	19% (645)	19% (493)	10% (50)	
Sum of Depression Item Scores	<2	49% (12004)	47% (8982)	52% (1574)	52% (1230)	59% (218)	<0.0001
	≥2	51% (12717)	53% (9879)	48% (1516)	48% (1165)	41% (157)	

Mean +/− SE (*N*) or % (*N*).

**Table 3:** Multivariable Associations of Nut and Walnut Consumption versus No Nut Consumption with Depression Scores.

		No Nuts	Other Nuts (ref = No Nuts)		Walnuts with Other Nuts (ref = No Nuts)		Walnuts with High Certainty (ref = No Nuts)	
	Sex	LS Mean (95% CL)	LS Mean (95% CL)	***p***	LS Mean (95% CL)	***p***	LS Mean (95% CL)	***p***
Sum of Depression Item Scores *	All	4.15(3.96, 4.34)	**3.83(3.6, 4.06)**	0.0028	**3.82(3.59, 4.06)**	0.0037	**3.28(2.85, 3.71)**	<0.0001
	Female	4.66(4.44, 4.87)	**4.14(3.87, 4.40)**	0.0001	**4.18(3.93, 4.43)**	0.0007	**3.54(3.08, 4.00)**	<0.0001
	Male	3.65(3.44, 3.86)	3.53(3.21, 3.84)	0.4604	3.46(3.14, 3.78)	0.2564	3.02(2.38, 3.66)	0.0533
			**OR (95%CL)**	***p***	**OR (95%CL)**	***p***	**OR (95%CL)**	***p***
Little interest in doing things **	All		0.92(0.81, 1.04)	0.1716	0.86(0.73, 1.02)	0.0862	**0.48(0.30, 0.78)**	0.0033
	Female		0.90(0.78, 1.05)	0.1804	0.83(0.68, 1.01)	0.0579	**0.46(0.29, 0.72)**	0.0008
	Male		0.93(0.76, 1.14)	0.4587	0.90(0.72, 1.13)	0.3669	0.51(0.24, 1.09)	0.0825
Feeling down, depressed, or hopeless	All		0.95(0.84, 1.07)	0.3879	0.87(0.73, 1.03)	0.1143	**0.63(0.43, 0.93)**	0.0211
	Female		0.88(0.75, 1.03)	0.1171	0.76(0.62, 0.94)	0.0119	**0.58(0.35, 0.95)**	0.0308
	Male		1.02(0.84, 1.24)	0.8633	0.99(0.76, 1.29)	0.9532	0.69(0.35, 1.36)	0.2774
Trouble sleeping or sleeping too much	All		0.93(0.83, 1.05)	0.2482	0.98(0.85, 1.13)	0.7912	0.96(0.69, 1.34)	0.8151
	Female		0.89(0.79, 1.01)	0.0781	0.95(0.78, 1.14)	0.5596	1.02(0.70, 1.47)	0.9310
	Male		0.98(0.81, 1.18)	0.8051	1.02(0.85, 1.22)	0.8578	0.91(0.52, 1.59)	0.7372
Feeling tired or having little energy	All		0.96(0.87, 1.06)	0.4039	0.95(0.85, 1.07)	0.3966	0.74(0.55, 1.00)	0.0532
	Female		0.94(0.83, 1.08)	0.3880	0.92(0.79, 1.06)	0.2422	**0.69(0.50, 0.96)**	0.0259
	Male		0.97(0.82, 1.16)	0.7661	0.99(0.81, 1.21)	0.9306	0.8(0.47, 1.36)	0.4089
Poor appetite or overeating	All		0.86(0.73, 1.01)	0.0725	0.87(0.75, 1.01)	0.0696	1.00(0.67, 1.49)	0.9830
	Female		**0.79(0.67, 0.94)**	0.0070	**0.83(0.70, 0.98)**	0.0290	0.77(0.51, 1.18)	0.2308
	Male		0.94(0.72, 1.21)	0.6153	0.91(0.71, 1.16)	0.4516	1.28(0.62, 2.64)	0.4971
Feeling bad about yourself	All		1.06(0.92, 1.22)	0.4216	1.02(0.88, 1.19)	0.7550	0.68(0.42, 1.10)	0.1123
	Female		0.97(0.81, 1.17)	0.7460	1.09(0.88, 1.34)	0.4267	0.63(0.39, 1.02)	0.0589
	Male		1.16(0.92, 1.46)	0.2178	0.97(0.76, 1.23)	0.7731	0.73(0.34, 1.54)	0.4036
Trouble concentrating on things	All		0.92(0.79, 1.06)	0.2495	0.93(0.78, 1.11)	0.4141	**0.53(0.31, 0.90)**	0.0194
	Female		0.86(0.71, 1.03)	0.1050	0.95(0.76, 1.19)	0.6593	0.70(0.46, 1.09)	0.1105
	Male		0.98(0.77, 1.25)	0.8732	0.91(0.69, 1.19)	0.4712	0.40(0.14, 1.11)	0.0781
Moving or speaking slowly or too fast	All		0.84(0.68, 1.03)	0.0993	0.86(0.65, 1.13)	0.2793	**0.51(0.28, 0.95)**	0.0342
	Female		0.81(0.63, 1.04)	0.0954	0.73(0.53, 1.01)	0.0575	**0.46(0.23, 0.94)**	0.0344
	Male		0.87(0.63, 1.21)	0.4054	1.01(0.68, 1.52)	0.9452	0.57(0.18, 1.80)	0.3310
Thought you would be better off dead	All		0.82(0.59, 1.15)	0.2459	**0.46(0.30, 0.70)**	0.0005	**0.13(0.04, 0.44)**	0.0016
	Female		0.68(0.47, 1.00)	0.0487	**0.46(0.27, 0.80)**	0.0064	**0.15(0.03, 0.65)**	0.0119
	Male		0.99(0.58, 1.70)	0.9779	**0.45(0.24, 0.86)**	0.0164	**0.11(0.01, 0.79)**	0.0284
Sum of Depression Item Scores (≥2)	All		0.92(0.82, 1.02)	0.1134	0.94(0.82, 1.07)	0.3194	**0.72(0.52, 1.00)**	0.0520
	Female		0.90(0.79, 1.03)	0.1169	0.94(0.78, 1.12)	0.4635	0.72(0.50, 1.04)	0.0823
	Male		0.93(0.79, 1.10)	0.4090	0.93(0.78, 1.13)	0.4739	0.73(0.43, 1.23)	0.2275
Sum of Depression Item Scores (≥5)	All		0.86(0.74, 1.00)	0.0535	**0.83(0.70, 0.98)**	0.0286	**0.39(0.22, 0.71)**	0.0022
	Female		0.85(0.71, 1.00)	0.0560	0.89(0.74, 1.07)	0.2289	**0.48(0.29, 0.78)**	0.0038
	Male		0.87(0.69, 1.11)	0.2593	**0.77(0.60, 1.00)**	0.0479	**0.32(0.13, 0.83)**	0.0199
Sum of Depression Item Scores (≥10)	All		0.80(0.64, 1.00)	0.0503	**0.67(0.48, 0.93)**	0.0185	0.51(0.22, 1.21)	0.1250
	Female		0.77(0.58, 1.01)	0.0617	**0.62(0.46, 0.84)**	0.0022	**0.46(0.22, 0.99)**	0.0459
	Male		0.83(0.56, 1.23)	0.3530	0.72(0.41, 1.27)	0.2522	0.56(0.11, 2.82)	0.4801

Data is from NHANES 2005–2014. Models adjusted for Age, Sex, Race, BMI, Smoking, Alcohol Use, Income, Marital Status, and Interaction between Walnut Consumption and Sex. * Sum of PHQ-9 questions DPQ010–090: Regression model, least square (LS) means are reported. ** Individual item: Logistic regression model, each item is dichotomized into no (0) and some depression symptom (1, 2, or 3). ORs = Odds rations of having depression symptom. Bolded numbers indicate statistically significant results whereas *p* < 0.05.

**Table 4:** Multivariable Associations of Walnut Consumption versus Other Nut Consumption with Depression Scores.

		Other Nuts	No Nuts (ref = Other Nuts)		Walnuts with Other Nuts (ref = Other Nuts)		Walnuts with High Certainty (ref = Other Nuts)	
	Sex	LS Mean (95% CL)	LS Mean (95% CL)	***p***	LS Mean (95% CL)	***p***	LS Mean (95% CL)	***p***
Sum of DPQ010-090 *	All	3.83(3.6, 4.06)	**4.15(3.96, 4.34)**	0.0028	3.82(3.59, 4.06)	0.9407	**3.28(2.85, 3.71)**	0.0134
	Female	4.14(3.87, 4.4)	**4.66(4.44, 4.87)**	0.0001	4.18(3.93, 4.43)	0.7448	**3.54(3.08, 4.00)**	0.0154
	Male	3.53(3.21, 3.84)	3.65(3.44, 3.86)	0.4604	3.46(3.14, 3.78)	0.7287	3.02(2.38, 3.66)	0.1394
			OR (95% CL) **	***p***	**OR (95% CL)**	***p***	**OR (95% CL)**	***p***
Little interest in doing things	All		1.09(0.96, 1.24)	0.1716	0.94(0.78, 1.14)	0.5404	**0.53(0.33, 0.85)**	0.0093
	Female		1.11(0.95, 1.28)	0.1804	0.91(0.74, 1.13)	0.3980	**0.51(0.32, 0.8)**	0.0043
	Male		1.08(0.88, 1.32)	0.4587	0.97(0.72, 1.31)	0.8490	0.55(0.25, 1.19)	0.1263
Feeling down, depressed, or hopeless	All		1.06(0.93, 1.20)	0.3879	0.92(0.75, 1.12)	0.4016	**0.67(0.45, 1.00)**	0.0488
	Female		1.13(0.97, 1.33)	0.1171	0.87(0.69, 1.08)	0.1966	0.66(0.39, 1.10)	0.1077
	Male		0.98(0.81, 1.20)	0.8633	0.98(0.71, 1.34)	0.8766	0.68(0.33, 1.39)	0.2868
Trouble sleeping or sleeping too much	All		1.07(0.95, 1.20)	0.2482	1.05(0.87, 1.27)	0.6134	1.03(0.72, 1.47)	0.8773
	Female		1.12(0.99, 1.27)	0.0781	1.06(0.85, 1.32)	0.6126	1.14(0.79, 1.64)	0.4937
	Male		1.02(0.85, 1.23)	0.8051	1.04(0.81, 1.34)	0.7556	0.93(0.51, 1.69)	0.8121
Feeling tired or having little energy	All		1.04(0.94, 1.15)	0.4039	0.99(0.86, 1.14)	0.9362	0.77(0.57, 1.05)	0.1010
	Female		1.06(0.93, 1.21)	0.3880	0.97(0.81, 1.17)	0.7550	0.73(0.53, 1.00)	0.0525
	Male		1.03(0.86, 1.22)	0.7661	1.02(0.78, 1.33)	0.8963	0.82(0.48, 1.43)	0.4822
Poor appetite or overeating	All		1.16(0.99, 1.37)	0.0725	1.01(0.84, 1.21)	0.9170	1.16(0.75, 1.79)	0.5055
	Female		**1.27(1.07, 1.50)**	0.0070	1.05(0.85, 1.29)	0.6591	0.98(0.63, 1.53)	0.9254
	Male		1.07(0.82, 1.38)	0.6153	0.97(0.72, 1.31)	0.8562	1.37(0.62, 3.01)	0.4323
Feeling bad about yourself	All		0.94(0.82, 1.09)	0.4216	0.97(0.8, 1.17)	0.7274	0.64(0.39, 1.05)	0.0767
	Female		1.03(0.86, 1.24)	0.7460	1.12(0.87, 1.44)	0.3737	0.65(0.40, 1.06)	0.0854
	Male		0.87(0.69, 1.09)	0.2178	0.84(0.64, 1.09)	0.1841	0.63(0.29, 1.39)	0.2495
Trouble concentrating on things	All		1.09(0.94, 1.27)	0.2495	1.01(0.83, 1.24)	0.8927	*0.58(0.33, 1.01)*	0.0547
	Female		1.17(0.97, 1.41)	0.1050	1.11(0.85, 1.45)	0.4272	0.82(0.51, 1.32)	0.4096
	Male		1.02(0.80, 1.30)	0.8732	0.92(0.68, 1.26)	0.6171	0.41(0.14, 1.16)	0.0907
Moving or speaking slowly or too fast	All		1.19(0.97, 1.47)	0.0993	1.02(0.72, 1.45)	0.8896	0.61(0.32, 1.17)	0.1355
	Female		1.24(0.96, 1.59)	0.0954	0.90(0.59, 1.38)	0.6335	0.57(0.27, 1.23)	0.1498
	Male		1.15(0.83, 1.58)	0.4054	1.16(0.69, 1.95)	0.5672	0.65(0.2, 2.13)	0.4720
Thought you would be better off dead	All		1.21(0.87, 1.69)	0.2459	**0.56(0.35, 0.88**)	0.0128	**0.15(0.04, 0.57)**	0.0057
	Female		**1.46(1.00, 2.13)**	0.0487	0.68(0.36, 1.28)	0.2265	0.22(0.05, 1.01)	0.0518
	Male		1.01(0.59, 1.72)	0.9779	**0.46(0.24, 0.88)**	0.0188	**0.11(0.01, 0.87)**	0.0369
Sum of DPQ010-090(≥2)	All		1.09(0.98, 1.21)	0.1134	1.02(0.86, 1.21)	0.8261	0.79(0.57, 1.1)	0.1595
	Female		1.11(0.97, 1.26)	0.1169	1.04(0.83, 1.29)	0.7403	0.8(0.54, 1.18)	0.2597
	Male		1.07(0.91, 1.26)	0.4090	1.00(0.77, 1.29)	0.9903	0.78(0.45, 1.33)	0.3561
Sum of DPQ010-090(≥5)	All		1.16(1.00, 1.35)	0.0535	0.96(0.79, 1.18)	0.7234	**0.46(0.25, 0.84)**	0.0120
	Female		1.18(1.00, 1.40)	0.0560	1.06(0.85, 1.31)	0.6155	**0.57(0.34, 0.95)**	0.0312
	Male		1.14(0.90, 1.45)	0.2593	0.88(0.64, 1.21)	0.4269	**0.37(0.14, 1.00)**	0.0510
Sum of DPQ010-090(≥10)	All		**1.25(1.00, 1.57)**	0.0503	0.84(0.59, 1.19)	0.3105	0.64(0.26, 1.58)	0.3271
	Female		1.3(0.99, 1.72)	0.0617	0.81(0.55, 1.18)	0.2632	0.6(0.27, 1.36)	0.2175
	Male		1.2(0.81, 1.78)	0.3530	0.86(0.47, 1.58)	0.6317	0.68(0.13, 3.58)	0.6429

Data is from NHANES 2005–2014. Models adjusted for Age, Sex, Race, BMI, Smoking, Alcohol Use, Income, Marital Status, and Interaction between Walnut Consumption and Sex. * Sum of PHQ-9 questions DPQ010-090: Regression model, LS means are reported. ** Individual item: Logistic regression model, each item is dichotomized into no (0) and some depression symptom (1, 2, or 3). ORs = Odds rations of having depression symptom. Bolded numbers indicate statistically significant results whereas *p* < 0.05.

## Abbreviations

NHANES	National Health and Nutrition Examination Survey
WwHC	walnuts with high certainty
WwON	walnuts with other nuts
ON	other nuts
NN	no nuts
OR	odds ratio
PHQ-9	Patient Health Questionnaire
